# 

**Published:** 1862-10

**Authors:** 


### CONTENTS.

### ORIGINAL CONTRIBUTIONS.

ART. XXXI. PROF. MAHLA'S REPORT ON THE CHIAGO RIVER,....... 587
ART. XXXII. Army Correspondence. E. ANDREWS, Surgeon of 1st Ill. Light Artillery, ........ 597
ART. XXXIII. A NEW SPLINT FOR FRACTURE OF THE FEMUR. By E. F. DODGE, M.D., Janesville, Wis. ........ 601
ART. XXXIV. GENERAL PATHOLOGY OF ERUPTIVE FEVERS, AND THE PRINCIPLES THAT SHOULD GOVERN THEIR TREATMENT. By N. S. DAVIS, M.D., ........ 604

### SELECTIONS.

On the Alkaloids of Hydrastis Canadensis. By Wm. S. Merrill, M.D., 609
Experimental Researches into a new Excretory Function of the Liver; Consisting in the Removal of Cholesterine from the Blood, and its Discharge from the Body in the form of Stercorine. (*The Seroline of Boudet.*) By Austin Flint, Jr., M.D., Professor of Physiology and Microscopy in the Bellevue Medical College, New York; &c... 617
Poisoning from the Pollen of the Common Yellow Tiger Lily. Read by Dr. Jeffries Wyman, ........ 631
Narcotics. By Joseph Bates, M.D. Atropa Belladonna........ 632
On the Value of Urinary Analysis in the Diagnosis and Treatment of Hepatic Disease. By George Harley, M.D., Prof. in University College, London,........ 641
Preservation of Animal Food,........ 643
The Astley Cooper Prize, ........ 644

### BOOK NOTICES.

The Hospital Steward's Manual; &c. By Joseph Janvier Woodward, M.D., Assistant-Surgeon U.S.A., &c. ........ 645
Anatomy of the Arteries of the Human Body, &c. By John Hatch Power, M.D., &c. ........ 645
Physician's Visiting List, for 1863. Published by Lindsay & Blakeston, of Philadelphia, ........ 645

### EDITORIAL.

N. Y. Ophthalmic School,........ 646
Illinois State Medical Society,........ 647
Chicago Medical Society, ........ 648
Appointment of Daniel Brainard, M.D., ........ 648
Chicago Medical Society, Monthly Meeting, ........ 648
Affections of the Throat in Scotland, ........ 649

